# Psychometric evaluation of a German version of the Muscularity-Oriented Eating Test (MOET) in men and women

**DOI:** 10.1186/s40337-026-01661-3

**Published:** 2026-05-30

**Authors:** Fabián Antonio Slama, Emilio J. Compte, Stuart B. Murray, Babette L. Winter, Malte Christian Claussen, Robin Halioua

**Affiliations:** 1 Sports Psychiatry Research Group, Center for Psychiatric Research, Department of Adult Psychiatry and Psychotherapy, University Hospital of Psychiatry Zurich, University of Zurich, Lenggstrasse 31, CH-8032 Zurich, Switzerland; 2 Eating Behavior Research Center, School of Psychology, Universidad Adolfo Ibáñez, Santiago, Chile; 3 Research Department, Comenzar de Nuevo, Monterrey, Mexico; 4 Department of Psychiatry and Biobehavioral Sciences, University of California Los Angeles, Los Angeles, CA USA; 5 Semel Institute for Neuroscience and Human Behavior, University of California Los Angeles, Los Angeles, CA USA; 6 Experimental Pharmacopsychology and Psychological Addiction Research, Center for Psychiatric Research, Department of Adult Psychiatry and Psychotherapy, University Hospital of Psychiatry Zurich, University of Zurich, Zurich, Switzerland; 7 Department of Public and Global Health, Epidemiology, Biostatistics and Prevention Institute, University of Zurich, Zurich, Switzerland; 8 Clinic for Depression and Anxiety, Psychiatric Centre Muensingen, Muensingen, Switzerland; 9 Praxis Liebestrasse, Winterthur, Switzerland

**Keywords:** Muscularity-oriented disordered eating, Muscularity-Oriented Eating Test, Muscle dysmorphia, Eating disorder screening, Psychometrics

## Abstract

**Background:**

Muscularity-oriented disordered eating (MODE) refers to eating behaviors that focus on muscularity rather than thinness. To assess this construct, the Muscularity-Oriented Eating Test (MOET) was developed and validated in several languages. This study aimed to translate and validate a German version of the MOET and to examine its factor structure and psychometric properties in physically active men and women.

**Methods:**

A total of 5,991 participants (5,556 women, 435 men) were recruited via Instagram followers of German-speaking fitness influencers. Demographic, exercise, and questionnaire data were collected. Factor structure was examined using exploratory and confirmatory factor analyses. Measurement invariance across gender, internal consistency, and convergent validity with related constructs were also assessed.

**Results:**

Among the previously proposed models, the original 15-item one-factor model showed acceptable fit in women but not in men, whereas the 12-item one-factor model showed acceptable fit in both gender groups. Allowing correlated residuals between theoretically related items further improved fit for these models. Exploratory analyses identified alternative solutions, including a 6-item one-factor model with good fit in both men and women. This model also demonstrated partial measurement invariance across gender. Across model versions, internal consistency was acceptable to good, and MOET scores showed positive associations with thinness-oriented eating pathology and muscle dysmorphia symptoms.

**Conclusions:**

The findings suggest that the German MOET is a useful instrument for assessing MODE. At the same time, the highly fitness-oriented composition of our sample may have contributed to the differences between our findings and those of previous validation studies. Further research in other populations, including clinical samples, is needed to further validate the assessment of MODE.

**Supplementary Information:**

The online version contains supplementary material available at 10.1186/s40337-026-01661-3.

## Abstract (plain language summary)

Some people try to become more muscular and change what and how they eat to reach that goal. For some, these eating habits can become unhealthy. The Muscularity-Oriented Eating Test (MOET) is a questionnaire that was first developed in English to measure eating-related thoughts and behaviors focused on muscularity rather than thinness. In this study, we translated the MOET into German and checked how well it works. Nearly 6,000 physically active women and men were recruited via German-speaking fitness influencers on Instagram and completed the German MOET and other questionnaires online. The original 15-item version worked well in our female sample, whereas in our male sample a previously tested 12-item version and a 6-item version developed in this study performed better. These two shorter versions also worked well in women. Across all versions, people with higher MOET scores also tended to report more muscle dysmorphia–related problems as well as more thinness-focused eating problems. The German MOET may be useful in future studies and as a screening tool in German-speaking populations. It is not a diagnostic tool, but it may help identify individuals who could benefit from further assessment.

**Supplementary Information:**

The online version contains supplementary material available at 10.1186/s40337-026-01661-3.

## Background

Muscularity-oriented disordered eating (MODE) refers to abnormal eating behaviors and attitudes specifically aimed at increasing muscle mass and achieving a more muscular, lean physique [1]. Unlike thinness-oriented eating disorders, which typically focus on weight loss [2], MODE is characterized by excessive concerns about protein intake, strict control of dietary practices, and the use of supplements or intentional overeating to increase muscle growth.

MODE may be accompanied or driven by muscle dysmorphia (MD), a subtype of body dysmorphic disorder [3, 4]. MD is primarily characterized by a chronic preoccupation with being insufficiently muscular and was first described by Pope, who referred to it as “reverse anorexia” [5]. While Pope et al. noted similarities between MD and eating disorders (EDs), they highlighted that individuals with MD primarily pursue muscularity through exercise rather than diet, distinguishing it from EDs. However, this classification has faced criticism, as disordered eating behaviors are often associated with MD and may reflect a male perspective within ED pathology [6]. From a transdiagnostic perspective, exercise and eating behaviors can be seen as two sides of the same coin, varying according to the desired aesthetic ideal. In the context of a muscular ideal, exercise is used to build muscle, while eating behaviors are modified to either promote muscle gain or reduce fat and achieve leanness. Both behaviors reflect body-modifying practices aimed at achieving the muscular aesthetic ideal, complementing each other in their effects.

MODE is not adequately assessed by tools designed for traditional, thinness-oriented eating behaviors [7]. To address this limitation, the Muscularity-Oriented Eating Test (MOET) was developed in 2019 [3]. Comprising 15 items, the MOET specifically evaluates muscularity-oriented eating behaviors, capturing attitudinal, affective, and behavioral aspects related to increasing muscle mass or reducing body fat. The original study, conducted with 519 male students from two U.S. universities, demonstrated good psychometric properties, including a one-factor structure and significant correlations with related constructs such as the Muscle Dysmorphic Disorder Inventory (MDDI), the Drive for Muscularity Scale (DMS), and the Eating Disorder Examination Questionnaire (EDE-Q).

Since then, the MOET has been translated and validated in multiple languages, including Spanish, Brazilian Portuguese, Chinese, and Turkish [8–11]. These studies replicated the original 15-item one-factor model and reported robust psychometric results. Although the original work focused on men, subsequent research extended the validation to women [10, 12, 13] and to specific populations, including gay men [14]. Whereas most studies supported the 15-item model, two studies tested a shorter 12-item one-factor version that demonstrated improved model fit among Australian undergraduate women [12], U.S. male and female athletes, and female non-athletes [15].

Recently, a German version of the MOET was examined in a mixed-gender sample, providing psychometric support for the 15-item one-factor model [16]. This work represents an important step toward establishing a German MOET, although additional evidence from different study contexts remains important, particularly from physically active populations in which MODE may be especially relevant. The present study extends previous work by examining the German MOET in a larger fitness-oriented sample and by evaluating its factor structure using gender-specific analyses.

The aim of the present study was to validate a German version of the MOET by testing the original 15-item and the shortened 12-item models and, if necessary, exploring alternative structures through exploratory and confirmatory factor analyses. We expected to replicate the original one-factor structure of the MOET and to find internal consistency comparable to that reported in previous studies. We further hypothesized that MOET scores would show significant positive correlations with muscle dysmorphia symptoms and thinness-oriented eating pathology.

## Methods

### Participants and design

This study is part of a larger investigation on body satisfaction, sports, and eating behaviors within a German-speaking online fitness community. Data were obtained in May 2021 using a cross-sectional design. Participants were recruited online through links shared on Instagram by three German-speaking male fitness influencers. Eligibility required an age of at least 18 years. After providing informed consent, participants completed anonymous online questionnaires (approx. 15 min) via REDCap [17]. No compensation was provided.

A total of 7,549 individuals (6,654 female, 515 male, 202 gender-diverse, and 178 did not report their gender) completed the survey. For the purposes of psychometric analyses, only male and female participants were considered, while gender-diverse and missing-gender participants were excluded due to small subgroup sizes. Final analytic sample sizes are reported in the Results section.

### Measures

#### Demographics

Participants reported age, gender, education, employment status, and marital status. Additionally, data on height and weight were collected, and participants were asked about sporting activities, including weekly hours of resistance training and other sports.

#### Muscularity-Oriented Eating Test (MOET)

The MOET [3] consists of 15 items assessing muscularity-oriented disordered eating (e.g., macronutrient tracking, supplement use, restrictive eating practices). Responses refer to the past 28 days and are rated on a 5-point Likert scale (0 = never to 4 = always). A higher mean score indicates more pronounced muscularity-oriented eating.

Because data for the present study were collected in 2021, this German MOET version was translated independently prior to the publication of the recently reported German adaptation. The translation followed a back-translation procedure [18]. The original English items were translated into German by one of the co-authors, a native German speaker with very good English proficiency, and subsequently back-translated into English by an independent individual who was a native speaker of both German and English and was not otherwise involved in the study. The back-translated version was then compared with the original English items, and discrepancies were resolved through revision of the German wording. In addition, feedback from the bodybuilding community was incorporated to ensure cultural and contextual adequacy. For this purpose, three individuals from the bodybuilding community were consulted independently, including two active competitive bodybuilders and one non-competitive natural bodybuilder. They were asked how they understood the items and whether they would endorse them. Based on this feedback, item 10 (“I have felt anxious when I run out of protein-based supplements”) was slightly adapted because the literal situation described in the original item was considered uncommon in this population and could therefore lead to false negative responses. Specifically, participants indicated that they would be unlikely to run out of protein supplements and would therefore tend to deny the item, even though they would feel anxious if this were to happen. The German wording therefore included an additional hypothetical element to reflect both the original meaning and a hypothetical scenario (corresponding to “I would have felt anxious if I had run out of protein-based supplements”). Although this constitutes a minor deviation from the original wording, the adaptation was intended to improve contextual relevance and better capture the rigidity of diet-related behavior in the target population. The original English items and adapted German items are provided in Supplementary Table S1.

#### Muscle Dysmorphic Disorder Inventory (MDDI)

The 13-item MDDI [19] assesses muscle dysmorphia pathology in three subscales: drive for size (DFS), functional impairment (FI), and appearance intolerance (AI). We used the validated German version [20], which showed good internal consistency (Cronbach’s α = 0.75). In our sample, internal consistency was modest (McDonald’s ω = 0.63).

#### Drive for thinness (DFT)

The German 7-item Drive for Thinness is a subscale of the Eating Disorder Inventory (EDI) [21], based on the original English version [22] and assesses excessive preoccupation with dieting and fear of weight gain on a 6-point Likert scale. Higher scores indicate a higher drive for thinness. In our sample, internal consistency was excellent (McDonald’s ω = 0.92).

#### Eating disorder examination questionnaire – 8-item version (EDE-Q8)

The 8-item German EDE-Q is a validated questionnaire designed to measure eating disorder psychopathology [23, 24]. The German version showed high internal consistency (Cronbach’s α = 0.93). The scale assesses symptoms over the past 28 days, with responses provided on a 7-point Likert scale. Higher scores indicate more severe eating disorder pathology. In our sample, internal consistency was also good (McDonald’s ω = 0.88).

### Data analysis

Data analyses followed general recommendations for test validation [25] and were conducted in R (version 4.4.0) using the packages *psych* [26] and *lavaan* [27]. For each gender, participants were randomly divided into two equally sized subsamples for exploratory and confirmatory analyses. Within each subsample, individuals with missing MOET item data were excluded prior to factor analyses.

In the first step, we tested the original 15-item and the reduced 12-item MOET models using confirmatory factor analyses (CFA) in the respective male and female subsamples. This approach allowed us to examine whether the previously established factor structures replicate in the German version. Because MOET items were assessed on ordered categorical response scales, CFAs were estimated using the weighted least squares mean and variance adjusted estimator (WLSMV), which is recommended for ordinal data. Model fit was evaluated using standard indices: chi-square (χ²), root mean square error of approximation (RMSEA), standardized root mean square residual (SRMR), comparative fit index (CFI), and Tucker–Lewis index (TLI). Acceptable model fit was defined as RMSEA ≤ 0.08, SRMR ≤ 0.08, CFI and TLI ≥ 0.90, with good fit indicated by RMSEA ≤ 0.05, SRMR ≤ 0.05, and CFI and TLI ≥ 0.95 [28]. Where theoretically justifiable and supported by modification indices, additional CFA models with correlated residuals were examined.

After testing the predefined models, exploratory factor analyses (EFA) were conducted to examine the underlying structure of the German MOET and to identify potential alternative models. EFAs were performed separately for the male and female EFA subsamples using principal axis factoring (PAF), as multivariate normality was violated (Mardia’s and Henze–Zirkler’s tests). Sampling adequacy was evaluated via Kaiser–Meyer–Olkin (KMO > 0.60) and Bartlett’s test of sphericity (*p* < .001). Factor retention was guided by parallel analysis, and oblimin rotation was applied given the expected factor correlations. Items were removed if factor loadings were < 0.30 or if cross-loadings occurred (primary > 0.40, secondary > 0.25).

In the final step, the alternative models identified in the EFAs were cross-validated using CFA in the independent confirmatory subsamples for men and women. Measurement invariance across gender was subsequently tested for the common 6-item MOET using nested multi-group CFA models. Specifically, configural, metric, and scalar invariance were examined by imposing increasing equality constraints across groups. Model comparisons were evaluated using changes in approximate fit indices, with invariance accepted when ΔCFI < 0.010 and ΔRMSEA < 0.015 [29].

Internal consistency of the MOET was primarily assessed using McDonald’s ω, with Cronbach’s α additionally reported descriptively where appropriate. Convergent validity was examined via Pearson correlations between MOET scores and related constructs (EDE-Q8, DFT, and MDDI, including its subscales). Effect sizes were interpreted according to Cohen’s guidelines [30], with *r* = .10 considered small, *r* = .30 moderate, and *r* = .50 large.

## Results

The final analytic sample consisted of 5,991 individuals (5,556 women and 435 men). After random splitting by gender for exploratory and confirmatory analyses, and exclusion of cases with missing MOET data, final EFA samples comprised 220 men and 2,778 women, and CFA samples 215 men and 2,778 women.

### Demographics and psychometrics

Demographic and psychometric data are shown in Table 1 for the male, female, and total samples. All values are based on the final analytic samples after exclusion of cases with missing MOET item data.

**Table 1:** Numeric and categorical demographic and psychometric characteristics for the male, female, and total study samples

Variable	Male (*n* = 435)	Female (*n* = 5,556)	Total (*n* = 5,991)
Numeric variables			
Age (years)	25.83 (6.22)	28.47 (7.16)	28.27 (7.13)
Body Mass Index (BMI, kg/m²)	26.12 (4.06)	25.67 (5.84)	25.70 (5.73)
Resistance training time (h/week)	6.26 (3.63)	2.66 (2.65)	2.92 (2.89)
Other sporting activity time (h/week)	3.10 (3.29)	3.16 (3.28)	3.16 (3.28)
EDE-Q8 mean	2.26 (1.45)	3.45 (1.48)	3.37 (1.51)
Drive for Thinness	19.11 (8.51)	28.75 (8.79)	28.05 (9.12)
MDDI total	34.96 (8.03)	30.15 (6.62)	30.50 (6.85)
Drive for size	14.53 (4.85)	8.53 (3.39)	8.97 (3.85)
Appearance intolerance	9.95 (3.81)	12.94 (3.85)	12.72 (3.93)
Functional impairment	10.48 (3.88)	8.68 (3.69)	8.81 (3.73)
MOET mean (15-item version)	1.29 (0.73)	1.22 (0.72)	1.22 (0.72)
MOET mean (12-item version)	1.34 (0.79)	1.38 (0.81)	1.38 (0.80)
MOET mean (female 10-item version)	–	1.24 (0.80)	–
MOET mean (6-item version)	1.23 (0.80)	1.03 (0.76)	1.04 (0.76)
Categorical variables			
Marital status			
Single	183 (42.1%)	1828 (32.9%)	2011 (33.6%)
Relationship	206 (47.4%)	2611 (47.0%)	2817 (47.0%)
Married	43 (9.9%)	1032 (18.6%)	1075 (17.9%)
Divorced	3 (0.7%)	81 (1.5%)	84 (1.4%)
Widowed	0 (0.0%)	4 (0.1%)	4 (0.1%)
Education			
No school diploma	5 (1.1%)	27 (0.5%)	32 (0.5%)
Secondary school	28 (6.4%)	165 (3.0%)	193 (3.2%)
Apprenticeship	112 (25.7%)	1171 (21.1%)	1283 (21.4%)
Qualifying for university admission	117 (26.9%)	1855 (33.4%)	1972 (32.9%)
Academic degree	173 (39.8%)	2338 (42.1%)	2511 (41.9%)
Employment status			
Apprenticeship	149 (34.3%)	1240 (22.3%)	1389 (23.2%)
Employee	201 (46.2%)	3503 (63.0%)	3704 (61.8%)
Senior Employee	44 (10.1%)	513 (9.2%)	557 (9.3%)
Self-employed	24 (5.5%)	140 (2.5%)	164 (2.7%)
Unemployed	17 (3.9%)	160 (2.9%)	177 (3.0%)
Sporting activity			
Bodybuilding or Resistance training	394 (90.6%)	3677 (66.2%)	4071 (68.0%)
Endurance	160 (36.8%)	3057 (55.0%)	3217 (53.7%)
Ballsports	84 (19.3%)	357 (6.4%)	441 (7.4%)
Martial Arts	24 (5.5%)	121 (2.2%)	145 (2.4%)
Track and field	6 (1.4%)	55 (1.0%)	61 (1.0%)
Gymnastics	6 (1.4%)	167 (3.0%)	173 (2.9%)
Other	60 (13.8%)	2251 (40.5%)	2311 (38.6%)

Values are presented as mean (SD) for numeric variables and as n (%) for categorical variables

Demographic variables and full 15-item MOET scores are based on participants with complete data for the 15-item MOET. Sample sizes for shorter MOET versions varied slightly because participants with missing responses on items not included in a given version could still be retained

– = not applicable

### Confirmatory factor analyses of the original 15-item one-factor model and the reduced 12-item one-factor model

The original 15-item one-factor model showed acceptable fit in the female CFA subsample, whereas fit in men was less satisfactory. The reduced 12-item one-factor model showed acceptable fit in both gender groups, with somewhat better fit indices in women than in men (Table 4). Because the male CFA subsample was relatively small (*n* = 215), we additionally tested the original 15-item model in the full male sample (*n* = 435). However, this did not materially change the overall pattern of results.

**Fig. 1:**
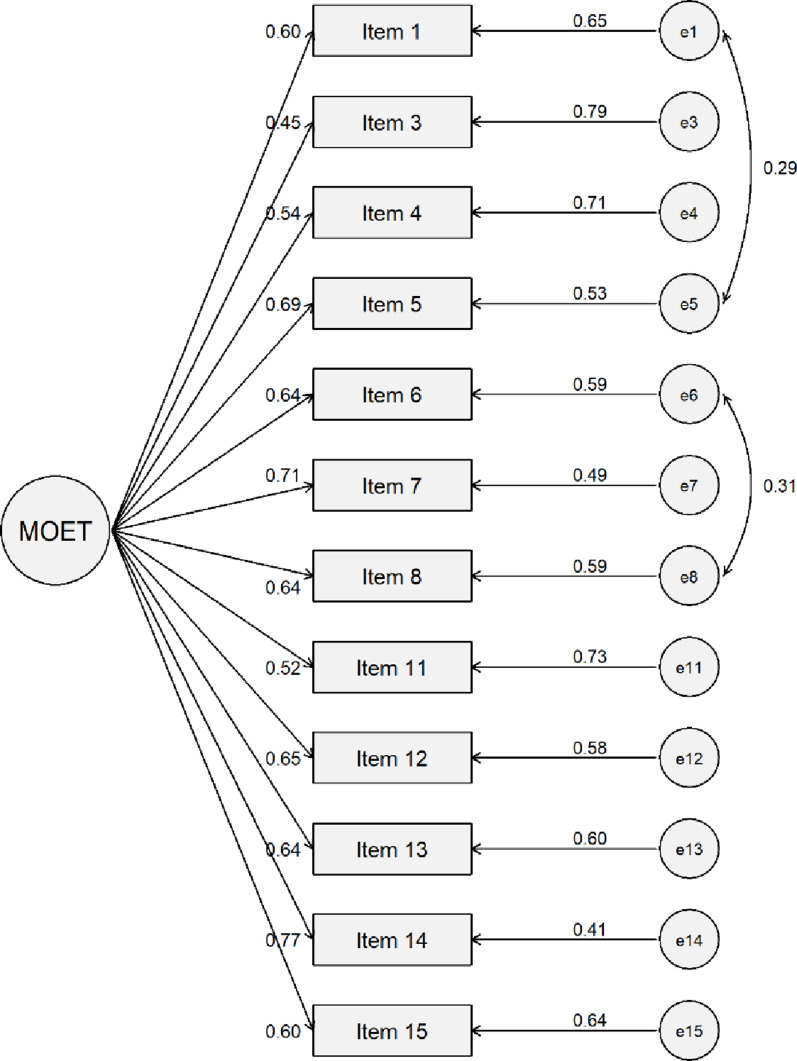
Confirmatory factor analysis of the male 12-item one-factor MOET model. Standardized factor loadings and residual variances are shown. The model included correlated residuals between Items 1 and 5 and between Items 6 and 8, indicated by curved double-headed arrows

**Fig. 2:**
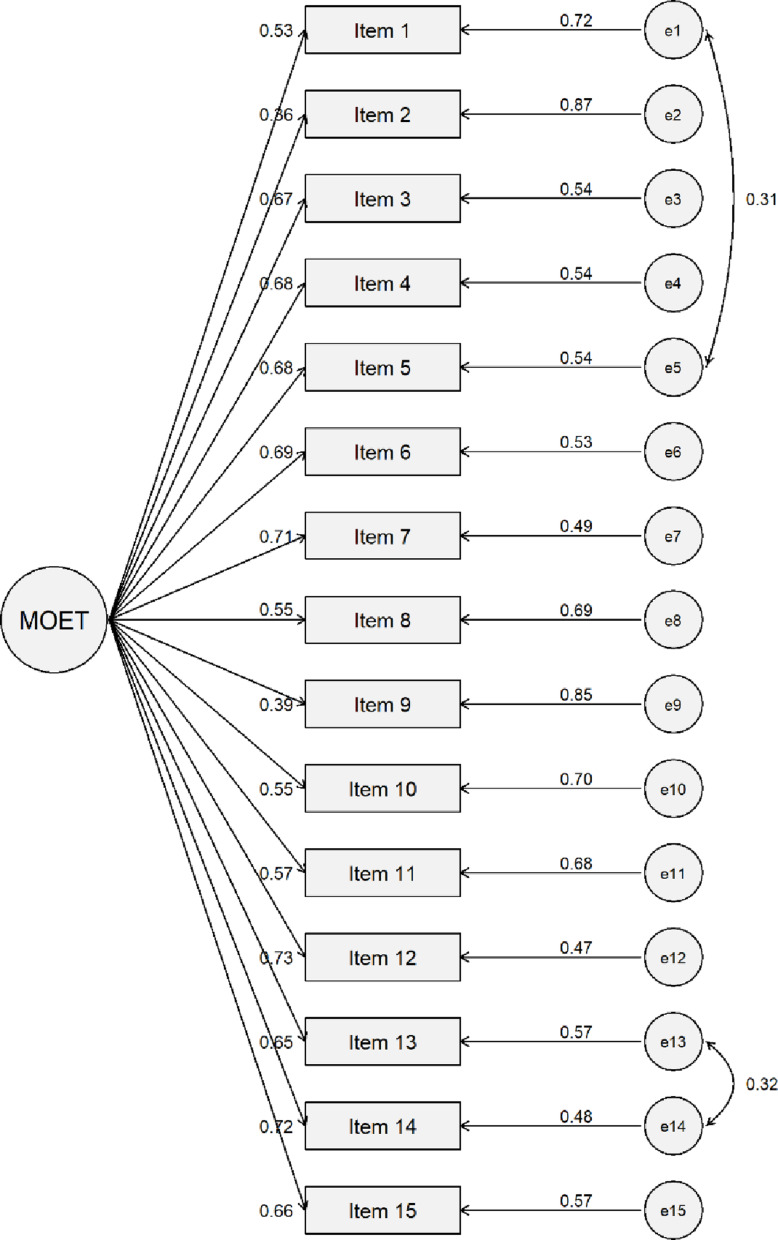
Confirmatory factor analysis of the female 15-item one-factor MOET model. Standardized factor loadings and residual variances are shown. The model included correlated residuals between Items 1 and 5 and between Items 13 and 14, indicated by curved double-headed arrows

Based on modification indices and guided by theoretical considerations, additional CFA models with correlated residuals were examined for conceptually related item pairs. In both gender groups, a residual correlation between Items 1 and 5 was considered theoretically justifiable because both refer to monitoring the macronutritional content of food in order to maintain dietary control. In men, modification indices additionally suggested residual correlations between Items 6 and 8, which were considered conceptually related because both describe advance preparation or planning of food intake to avoid deviating from a diet plan. Allowing residual correlations between these item pairs did not meaningfully improve fit of the 15-item model and was therefore not pursued further. However, applying the same modifications to the 12-item model resulted in better fit indices (Table 4; Fig. 1). In women, modification indices instead suggested a residual correlation between Items 13 and 14, which were considered conceptually related because both refer to concerns about how one’s diet-related rules are viewed or understood by others. Allowing residual correlations between Items 1 and 5 and between Items 13 and 14 improved fit of the 15-item model (Table 4; Fig. 2).

Standardized factor loadings were generally moderate to high across all one-factor models (see Supplementary Table S3).

To further investigate the factor structure of the German MOET in our sample, exploratory factor analyses were conducted in the independent EFA subsamples.

### Exploratory factor analyses

In all EFAs, sampling adequacy was confirmed (KMO > 0.60; Bartlett’s test *p* < .001).

#### Men (EFA subsample, *n* = 220)

In the male EFA subsample, the initial 15-item analysis suggested a two-factor solution according to parallel analysis. Item 2 showed an insufficient factor loading, whereas items 5, 8, 9, 12, and 14 showed problematic cross-loadings. These items were therefore removed before the next analysis. The subsequent 9-item EFA suggested a three-factor solution.

Although item 7 did not formally exceed the predefined cross-loading threshold, it was excluded due to its ambiguous loading pattern and elevated item complexity (com = 2.2). In the following 8-item solution, items 4 and 13 showed ultra-Heywood cases (h² > 1), indicating model inadmissibility, and were removed. The final 6-item EFA supported a one-factor solution, with all retained items showing adequate loadings. However, mean communality was relatively low and uniqueness was high, indicating that the extracted factor explained only a limited proportion of item variance. Detailed item-level statistics for the final 6-item one-factor solution are shown in Table 2.

**Table 2:** Item statistics for the final male EFA model

	F1	h2	u2	com
Item 1	0.53	0.28	0.72	1
Item 3	0.45	0.20	0.80	1
Item 6	0.74	0.55	0.45	1
Item 10	0.46	0.21	0.79	1
Item 11	0.47	0.22	0.78	1
Item 15	0.60	0.36	0.64	1

Factor loadings (pattern matrix) from the final exploratory factor analyses

*F1* = extracted factor, *h2* communality, *u2* uniqueness, *com* item complexity

#### Women (EFA subsample, *n* = 2,778)

In the female EFA subsample, the initial 15-item analysis suggested a six-factor solution according to parallel analysis. Item 2 was removed because of an insufficient factor loading, and item 4 was excluded because of problematic cross-loadings. The subsequent 13-item EFA again suggested a six-factor solution, but the model was inadmissible because item 12 showed an ultra-Heywood case and was therefore removed. In the following 12-item analysis, parallel analysis supported a five-factor solution. Items 8 and 9 showed insufficient factor loadings and were excluded. The final 10-item EFA resulted in a five-factor solution with two items per factor and adequate communalities. The resulting item pairings and statistics are shown in Table 3.

**Table 3:** Item statistics for the final female EFA model

	F1	F2	F3	F4	F5	h2	u2	com
Item 1	-0.01	0.00	**0.74**	-0.02	0.02	0.54	0.46	1
Item 3	0.01	**0.82**	-0.01	0.02	0.02	0.71	0.29	1
Item 5	0.11	0.07	**0.48**	0.11	0.05	0.47	0.53	1.3
Item 6	-0.03	0.01	0.03	0.00	**0.65**	0.43	0.57	1
Item 7	0.09	**0.45**	0.26	0.03	0.01	0.46	0.54	1.7
Item 10	0.15	-0.11	0.11	**0.31**	0.15	0.29	0.71	2.6
Item 11	-0.01	0.03	-0.01	**0.70**	-0.01	0.49	0.51	1
Item 13	**0.60**	0.17	-0.05	-0.04	0.00	0.46	0.54	1.2
Item 14	**0.75**	-0.05	0.06	0.04	0.02	0.61	0.39	1
Item 15	0.27	0.07	-0.07	0.05	**0.34**	0.34	0.66	2.2

*h2* communality, *u2* uniqueness, *com* item complexity

Factor loadings (pattern matrix) from the final exploratory factor analyses

F1–F5 = extracted factors

Highest factor loadings are bolded

### Confirmatory factor analyses of the new models

The alternative models identified in the EFAs were subsequently cross-validated in the independent CFA subsamples. The 6-item one-factor model demonstrated excellent fit in men, whereas the 10-item five-factor model showed good fit in women. The 6-item one-factor model was also tested in women to evaluate its suitability as a common short version across gender groups and likewise demonstrated good fit (Table 4). Standardized factor loadings are shown in Supplementary Figure S2 for the female 10-item five-factor model and in Supplementary Table S3 for the one-factor models. Because the 6-item model showed good fit in both men and women, it was considered suitable for measurement invariance testing across gender. Configural and metric invariance were supported, whereas full scalar invariance was not. After freeing the thresholds of Item 3, partial scalar invariance was achieved (see Supplementary Table S4).

**Table 4:** Fit indices for confirmatory factor analyses of the German MOET across model specifications and gender groups

Group	Model	Modification	*n*	χ²(df)	CFI	TLI	RMSEA [90% CI]	SRMR
Men	15-item, 1-factor		215	237.67 (90)	0.91	0.89	0.09 [0.07–0.10]	0.10
	12-item, 1-factor		215	140.42 (54)	0.94	0.92	0.09 [0.07–0.10]	0.08
	12-item, 1-factor	correlated residuals (Items 1 and 5; 6 and 8)	215	124.10 (52)	0.95	0.93	0.08 [0.06–0.10]	0.08
	6-item, 1-factor		215	5.02 (9)	1.00	1.03	0.00 [0.00–0.05]	0.03
Women	15-item, 1-factor		2,778	1,457.96 (90)	0.94	0.93	0.07 [0.07–0.08]	0.06
	15-item, 1-factor	correlated residuals (Items 1 and 5; 13 and 14)	2,778	1,120.53 (88)	0.96	0.95	0.07 [0.06–0.07]	0.05
	12-item, 1-factor		2,781	1,107.66 (54)	0.95	0.94	0.08 [0.08–0.09]	0.05
	10-item, 5-factor		2,781	278.75 (25)	0.98	0.97	0.06 [0.05–0.07]	0.04
	6-item, 1-factor		2,785	112.20 (9)	0.97	0.95	0.06 [0.05–0.08]	0.03

*CFI* Comparative Fit Index, *TLI* Tucker–Lewis Index, *RMSEA* Root Mean Square Error of Approximation, *SRMR* Standardized Root Mean Square Residual

All models were estimated in the gender-specific CFA subsamples; slight differences in n across models reflect case-wise exclusion due to missing item responses

Chi-square values (χ²) are reported for completeness but were not used to evaluate model fit due to their sensitivity to sample size

CFAs were estimated using the weighted least squares mean and variance adjusted estimator (WLSMV)

Cut-offs for acceptable model fit were RMSEA ≤ 0.08, SRMR ≤ 0.08, CFI ≥ 0.90, and TLI ≥ 0.90 (good fit: RMSEA ≤ 0.05, SRMR ≤ 0.05, CFI/TLI ≥ 0.95; Hu & Bentler, 1999)

RMSEA values are reported with 90% confidence intervals in brackets where applicable

### Reliability

In the male CFA subsample, internal consistency was good for the 15-item model (ω = 0.85, 95% CI [0.82, 0.88]) and the 12-item model (ω = 0.85, 95% CI [0.81, 0.88]), whereas the 6-item model showed lower but acceptable internal consistency (ω = 0.65, 95% CI [0.57, 0.71]). In the male EFA subsample, the final 6-item solution likewise showed acceptable internal consistency (ω = 0.70, 95% CI [0.60, 0.77]).

In the female CFA subsample, internal consistency was good for the 15-item model (ω = 0.87, 95% CI [0.86, 0.88]) and the 12-item model (ω = 0.87, 95% CI [0.86, 0.87]), whereas the 6-item model showed acceptable internal consistency (ω = 0.69, 95% CI [0.67, 0.71]). For the female 10-item five-factor model, Cronbach’s α coefficients for the two-item factors ranged from 0.50 to 0.70 in the CFA subsample, indicating limited to moderate internal consistency at the subscale level.

### Convergent validity

All MOET versions were positively associated with thinness-oriented eating pathology and muscle dysmorphia-related symptoms (Table 5).

In women, the 12-item and 15-item versions showed stronger correlations with thinness-oriented measures (EDE-Q8, DFT) than the 6-item version, whereas correlations with the MDDI and functional impairment (FI) were similarly high across versions. Correlations with appearance intolerance (AI) were also lower for the 6-item version, while associations with drive for size (DFS) remained low across all female MOET versions.

In men, a similar pattern emerged. The 12-item version showed the strongest correlations with EDE-Q8, DFT, and AI, whereas the 6-item version correlated less strongly with thinness-oriented measures. Correlations with the MDDI and FI remained high and comparable across versions. In contrast to women, correlations with DFS were higher in men and were highest for the 6-item version. All correlations are reported in Table 5.

**Table 5:** Correlations of muscularity-oriented eating test scores with other psychometric variables

	EDE-Q8	DFT	MDDI total	AI	DFS	FI
Female (15-item MOET)	0.57 [0.55, 0.59]	0.58 [0.57, 0.60]	0.55 [0.53, 0.57]	0.34 [0.32, 0.36]	0.07 [0.05, 0.10]	0.57 [0.55, 0.59]
Female (12-item MOET)	0.59 [0.58, 0.61]	0.61 [0.59, 0.62]	0.54 [0.52, 0.56]	0.35 [0.33, 0.38]	0.05 [0.03, 0.08]	0.55 [0.53, 0.57]
Female (6-item MOET)	0.46 [0.44, 0.48]	0.46 [0.44, 0.48]	0.51 [0.49, 0.53]	0.25 [0.23, 0.28]	0.09 [0.07, 0.12]	0.56 [0.54, 0.58]
Male (15-item MOET)	0.44 [0.37, 0.52]	0.32 [0.24, 0.41]	0.56 [0.49, 0.62]	0.29 [0.20, 0.38]	0.25 [0.16, 0.34]	0.55 [0.49, 0.62]
Male (12-item MOET)	0.51 [0.43, 0.57]	0.40 [0.32, 0.48]	0.52 [0.45, 0.59]	0.34 [0.26, 0.42]	0.19 [0.10, 0.28]	0.51 [0.44, 0.58]
Male (6-item MOET)	0.37 [0.29, 0.45]	0.25 [0.16, 0.33]	0.54 [0.47, 0.60]	0.22 [0.13, 0.31]	0.29 [0.20, 0.37]	0.53 [0.46, 0.60]

*EDE-Q8* Eating Disorder Examination–Questionnaire 8, *DFT* Drive for Thinness, *MDDI* Muscle Dysmorphic Disorder Inventory, *AI* Appearance Intolerance, *DFS* Drive for Size, *FI* Functional Impairment

Values are Pearson’s r (95% CI)

All p-values < 0.001

Sample sizes varied slightly across model versions due to missing data

For men, *n* = 435 for the 15-item MOET and *n* = 436 for the 12-item and 6-item MOET versions; for women, *n* = 5,556 for the 15-item MOET, *n* = 5,561 for the 12-item MOET, and *n* = 5,568 for the 6-item MOET

## Discussion

The aim of this study was to validate a German version of the Muscularity-Oriented Eating Test (MOET) and to examine its psychometric properties in men and women. Overall, the findings suggest that the original 15-item one-factor model showed acceptable fit in women, whereas in men the shorter 12-item one-factor model demonstrated better fit. In addition, exploratory analyses identified alternative models, including a brief 6-item version that showed good fit in both gender groups.

These findings are partly consistent with the growing international literature on the MOET. Several validation studies across different languages and populations have supported the original 15-item one-factor structure and reported overall good psychometric properties, often in student or broader community samples [8–11, 13]. At the same time, some studies have shown that a 12-item version may provide improved fit in specific populations, including women and athletic samples [12, 15]. The better fit of the 12-item model in our male sample may also partly reflect the specific participant composition, as recruitment took place in a highly fitness-oriented online community.

The comparison with a recently published German study is particularly informative. That study was based on a smaller mixed-gender sample (*n* = 525), which was also predominantly female (68.6%) [16]. Because the original 15-item model showed acceptable fit in their overall sample, the differences between the two studies may partly reflect the use of gender-specific rather than combined analyses, as well as differences in sample characteristics and possibly minor translation or adaptation differences. Internal consistency in our study was somewhat lower. Overall, these findings do not necessarily indicate a contradiction between the two studies, but rather underline the importance of study context and analytic strategy when evaluating a German MOET.

Regarding the alternative models identified in the exploratory analyses, the female 10-item five-factor solution suggests that MOET items may show some multidimensionality. This is broadly consistent with the original conceptualization of the MOET, which was intended to capture attitudinal, behavioural, and affective aspects of muscularity-oriented eating pathology rather than a narrowly defined single behaviour [3]. At the same time, this model is of greater theoretical than practical interest, as models with only two items per factor are psychometrically difficult to justify [31].

By contrast, the 6-item short version appears particularly relevant as a pragmatic common solution. The retained items capture core aspects of MODE, and its lower correlations with thinness-oriented measures, together with similarly high associations with the MDDI and functional impairment, suggest that it may capture a somewhat more specific muscularity-oriented component of eating pathology. However, some theoretically relevant features of the original scale are lost in the short form, including preparing meals in advance and continuing to eat despite feeling full. In addition, internal consistency was lower than for the longer versions, and Item 3 showed a weak loading in the male sample. Taken together, the short version may represent a useful brief alternative for specific research purposes, particularly when brevity and cross-gender comparability are important, but it would benefit from further validation.

Findings on convergent validity were largely consistent with previous studies and thus provide further support for the validity of the German MOET. At the same time, the pattern of associations across versions and gender groups helps to contextualize how muscularity-oriented eating relates to thinness-oriented pathology. The gender-specific pattern of associations suggests that muscularity-oriented eating may be linked more closely to a straightforward drive for size in men than in women. In women, the weak associations with drive for size may indicate either that MODE is tied to a more mixed body ideal involving thinness, leanness, and muscularity, or that the MOET does not capture muscularity-oriented eating equally well in women, particularly if some items are open to interpretation in the direction of thinness-related concerns. In contrast to the original study, our data also showed lower correlations with appearance intolerance but higher correlations with functional impairment, which may reflect the characteristics of our fitness-oriented sample.

Several limitations should be acknowledged.

First, unlike other validation studies, test–retest reliability was not assessed. Future research should therefore examine the temporal stability of the German MOET.

Second, although participants were followers of German-speaking fitness influencers, being a native German speaker was not an inclusion criterion. Therefore, we cannot fully rule out the possibility that some participants experienced linguistic difficulties with individual items.

Third, the translation process may not have fully met recommended guidelines for cross-cultural test adaptation, such as multiple independent forward and back translations [32], potentially introducing minor linguistic discrepancies.

Fourth, the study sample consisted of followers of fitness influencers, which limits generalizability to the broader population, even though it also represents a group in which muscularity-oriented eating concerns are likely to be especially relevant.

Fifth, all data were based on online self-report, which may have introduced reporting biases.

Finally, the large difference in sample sizes between women and men may have influenced the EFA results. Future studies with more balanced gender groups could clarify whether the observed differences are due to structural or sampling effects.

Despite these limitations, the present study provides important new evidence on the psychometric properties of an adapted German version of the MOET. It includes a large overall sample, focuses on a population at elevated risk for MODE, and combines exploratory and confirmatory approaches with measurement invariance testing. This made it possible not only to evaluate previously proposed models, but also to identify alternative structures that may be useful depending on the research context.

## Conclusions

In summary, the present findings suggest that the German MOET is a useful instrument for assessing muscularity-oriented disordered eating, although no single model performed equally well across all groups. The 6-item version may be useful when brevity and cross-gender comparability are priorities. Future studies should further examine the German MOET in other cultural contexts and clinical populations, clarify its longitudinal stability and practical screening utility, and examine whether shorter MOET versions can be replicated in other languages and populations.

## Supplementary Information

Below is the link to the electronic supplementary material.

Supplementary Material 1.

## Data Availability

The data are not publicly available due to participant confidentiality but are available from the corresponding author on reasonable request.

## Abbreviations

MODE: Muscularity-oriented disordered eating
MOET: Muscularity-Oriented Eating Test
ED: Eating disorder
EDE-Q: Eating disorder examination questionnaire
EDE-Q8: Eating disorder examination questionnaire – 8-item version
EDI: Eating Disorder Inventory
MD: Muscle dysmorphia
MDDI: Muscle dysmorphic disorder inventory
DFS: Drive for size
AI: Appearance intolerance
FI: Functional impairment
DFT: Drive for thinness
DMS: Drive for muscularity scale
EFA: Exploratory factor analysis
CFA: Confirmatory factor analysis
WLSMV: Weighted least squares mean and variance adjusted estimator
KMO: Kaiser–Meyer–Olkin measure
PAF: Principal axis factoring
SRMR: Standardized root mean square residual
RMSEA: Root mean square error of approximation
CFI: Comparative fit index
TLI: Tucker-Lewis index
