# Supplementary material for: Psychometric evaluation of a German version of the Muscularity-Oriented Eating Test (MOET) in men and women
Source: J Eat Disord. 2026 May 30;14:178. doi: 10.1186/s40337-026-01661-3 (PMC13440089; doi:10.1186/s40337-026-01661-3)
Supplement: Supplementary file 1 [file 40337_2026_1661_MOESM1_ESM.docx]

| **Supplementary Table S1. Original English and German MOET items with retention across model versions** | | | |
| --- | --- | --- | --- |
| **Item** | **Original English Items** | **German Items** | **Models retained** |
| **1** | I have recorded the macro-nutritional values of everything that I ate. | Ich habe die Makro-Nährwerte von allem, was ich gegessen habe, protokolliert. | 12, F10, 6 |
| **2** | I have used meal replacement supplements when I felt full. | Ich habe Mahlzeitenersatzpräparate verwendet, wenn ich mich satt fühlte. | – |
| **3** | What I ate has influenced how I think about myself as a person. | Was ich gegessen habe, hat Einfluss darüber gehabt, wie ich über mich als Person denke. | 12, F10, 6 |
| **4** | There are definite foods I have avoided eating due to worry about how they might affect my shape or weight. | Es gab bestimmte Lebensmittel, die ich aus Sorge darüber, wie sie sich auf meine Form oder mein Gewicht auswirken könnten, vermieden habe. | 12 |
| **5** | I have felt less anxious about eating out if I knew the macro-nutritional content of the food at the restaurant. | Ich hatte weniger Angst davor, auswärts zu essen, wenn ich den Makronährstoffgehalt des Essens im Restaurant kannte. | 12, F10 |
| **6** | I have taken my own food out with me to social events in case the food on offer is inconsistent with my diet plan. | Ich habe mein eigenes Essen zu gesellschaftlichen Anlässen mitgenommen, falls das angebotene Essen nicht mit meinem Ernährungsplan vereinbar gewesen wäre. | 12, F10, 6 |
| **7** | I cannot achieve my body ideal unless I exert complete control over everything I eat. | Ich kann mein Körperideal nicht erreichen, ohne die vollständige Kontrolle darüber zu haben, was ich esse. | 12, F10 |
| **8** | I have pre-cooked several meals in advance to ensure that I do not deviate from my diet plan. | Ich habe mehrere Mahlzeiten vorgekocht, um sicherzustellen, dass ich nicht von meinem Ernährungsplan abweiche. | 12 |
| **9** | I have continued eating despite feeling full, in attempting to influence my muscularity. | Um den Grad meiner Muskulosität zu beeinflussen, habe ich auch dann weiter gegessen, wenn ich schon satt war. | – |
| **10** | I have felt anxious when I run out of protein-based supplements. | Ich bin oder wäre beunruhigt gewesen, wenn mir meine Protein-Supplements ausgegangen sind oder wären. | F10, 6 |
| **11** | I have been deliberately trying to limit the overall volume of some foods, so that my muscles look more defined. | Ich habe bewusst versucht, die Menge einiger Lebensmittel zu begrenzen, damit meine Muskeln definierter aussehen. | 12, F10, 6 |
| **12** | If I broke any of my food rules, I attempted to make up for it at my next meal. | Wenn ich eine meiner Ernährungsregeln brach, habe ich versucht, dies bei der nächsten Mahlzeit zu kompensieren. | 12 |
| **13** | I have felt anxious about others knowing the rules I have around what I eat. | Ich war besorgt, dass andere von meinen Ernährungsregeln erfahren. | 12, F10 |
| **14** | Other people do not seem to understand how important my food choices are to me. | Andere Menschen scheinen nicht zu verstehen, wie wichtig mir die Wahl meiner Lebensmittel ist. | 12, F10 |
| **15** | Ensuring proper adherence to my dietary ideals is more important to me than adhering to a work schedule. | Das korrekte Befolgen und Umsetzen meiner Ernährungsvorstellungen waren mir wichtiger als das Einhalten von Arbeitszeiten. | 12, F10, 6 |
| **Note**. Response format: 0 = never true (*nie*), 1 = rarely true (*selten*), 2 = sometimes true (*manchmal*), 3 = usually true (*oft*), 4 = always true (*immer*). Reference period: past 28 days. The column “Models retained” indicates whether an item was retained in the 12-item one-factor model (“12”), the female 10-item five-factor model (“F10”), or the common 6-item one-factor model (“6”). | | | |

**Supplementary Figure S2: Confirmatory factor analysis of the female 10-item five-factor MOET model.**

**Note.** Standardized factor loadings and inter-factor correlations are shown.

| **Supplementary Table S3. Standardized factor loadings for the one-factor MOET models in men and women** | | | | | | | | |
| --- | --- | --- | --- | --- | --- | --- | --- | --- |
| Item | Men 15-item | Men 12-item | Men 12-item + RC | Men 6-item | Women 15-item | Women 15-item + RC | Women 12-item | Women 6-item |
| Item 1 | 0.63 | 0.63 | 0.60 | 0.59 | 0.56 | 0.53 | 0.55 | 0.51 |
| Item 2 | 0.34 | – | – | – | 0.36 | 0.36 | – | – |
| Item 3 | 0.44 | 0.45 | 0.46 | 0.27 | 0.67 | 0.68 | 0.68 | 0.55 |
| Item 4 | 0.52 | 0.53 | 0.54 | – | 0.67 | 0.68 | 0.68 | – |
| Item 5 | 0.70 | 0.72 | 0.69 | – | 0.70 | 0.68 | 0.71 | – |
| Item 6 | 0.69 | 0.68 | 0.64 | 0.72 | 0.68 | 0.69 | 0.67 | 0.71 |
| Item 7 | 0.71 | 0.71 | 0.71 | – | 0.71 | 0.71 | 0.72 | – |
| Item 8 | 0.68 | 0.68 | 0.64 | – | 0.55 | 0.55 | 0.54 | – |
| Item 9 | 0.39 | – | – | – | 0.39 | 0.39 | – | – |
| Item 10 | 0.60 | – | – | 0.56 | 0.54 | 0.55 | – | 0.60 |
| Item 11 | 0.50 | 0.52 | 0.52 | 0.53 | 0.56 | 0.57 | 0.55 | 0.58 |
| Item 12 | 0.66 | 0.64 | 0.65 | – | 0.72 | 0.73 | 0.72 | – |
| Item 13 | 0.59 | 0.63 | 0.64 | – | 0.69 | 0.65 | 0.70 | – |
| Item 14 | 0.76 | 0.76 | 0.77 | – | 0.75 | 0.72 | 0.75 | – |
| Item 15 | 0.61 | 0.59 | 0.60 | 0.65 | 0.65 | 0.66 | 0.65 | 0.68 |
| **Note.** Values represent standardized factor loadings from the respective CFA models. A dash (–) indicates that the item was not included in a given model. “+ RC” denotes models including theory-guided residual correlations. In men, the modified 12-item model included residual correlations between Items 1 and 5 and between Items 6 and 8. In women, the modified 15-item model included residual correlations between Items 1 and 5 and between Items 13 and 14. | | | | | | | | |

| **Supplementary Table S4: Measurement invariance of the 6-item MOET across gender groups** | | | | | | |
| --- | --- | --- | --- | --- | --- | --- |
| Model | CFI | TLI | RMSEA | SRMR | ΔCFI vs prev | ΔRMSEA vs prev |
| Configural | 0.976 | 0.959 | 0.059 | 0.037 | — | — |
| Metric | 0.977 | 0.969 | 0.051 | 0.041 | 0.001 | -0.008 |
| Scalar | 0.924 | 0.943 | 0.07 | 0.039 | -0.053 | 0.019 |
| Partial Scalar (Item 3 freed) | 0.974 | 0.978 | 0.043 | 0.039 | -0.003 | -0.008 |
| **Note**. Measurement invariance was tested using nested multi-group CFA models. CFI = Comparative Fit Index; TLI = Tucker–Lewis Index; RMSEA = Root Mean Square Error of Approximation; SRMR = Standardized Root Mean Square Residual. ΔCFI and ΔRMSEA are reported relative to the preceding model, except for partial scalar invariance, which is reported relative to the metric model. A decrease in CFI of less than .010 and an increase in RMSEA of less than .015 were considered evidence of invariance (Chen, 2007). Partial scalar invariance was achieved by freeing the thresholds of Item 3. | | | | | | |
